# Glycycoumarin inhibits hepatocyte lipoapoptosis through activation of autophagy and inhibition of ER stress/GSK-3-mediated mitochondrial pathway

**DOI:** 10.1038/srep38138

**Published:** 2016-11-30

**Authors:** Enxiang Zhang, Shutao Yin, Xinhua Song, Lihong Fan, Hongbo Hu

**Affiliations:** 1 Beijing Advanced Innovation Center for Food Nutrition and Human Health, College of Food Science and Nutritional Engineering, China Agricultural University, National Engineering Research Center for Fruit and Vegetable Processing, Beijing, China No. 17 Qinghua East Road, Haidian District, Beijing, 100083, China; 2 College of Veterinary Medicine, China Agricultural University, No. 2 Yunamingyuan West Road, Haidian District, Beijing, 100193, China

## Abstract

Herbal medicine as an alternative approach in the treatment of disease has drawn growing attention. Identification of the active ingredient is needed for effective utilization of the herbal medicine. Licorice is a popular herbal plant that is widely used to treat various diseases including liver diseases. Glycycoumarin (GCM) is a representative of courmarin compounds isolated from licorice. In the present study, the protective effect of GCM on hepatocyte lipoapoptosis has been evaluated using both cell culture model of palmitate-induced lipoapoptosis and animal model of non-alcoholic steatohepatitis (NASH). The results demonstrated for the first time that GCM was highly effective in suppressing hepatocyte lipoapoptosis in both *in vitro* and *in vivo*. Mechanistically, GCM was able to re-activate the impaired autophagy by lipid metabolic disorders. In line with the activation of autophagy, ER stress-mediated JNK and mitochondrial apoptotic pathway activation was inhibited by GCM both *in vitro* and *in vivo*. In addition, inactivation of GSK-3 might also contribute to the protective effect of GCM on hepatocyte lipoapoptosis. Our findings supported GCM as a novel active component of licorice against non-alcoholic fatty liver disease (NAFLD).

Non-alcoholic fatty liver disease (NAFLD) is the most common type of liver disease and accounts for nearly half of the total liver diseases worldwide. It is closely associated with a group of disorders, such as obesity and type 2 diabetes^1^. Although simple steatosis generally does not cause complications, a subset of patients with NAFLD will develop more serious liver injuries including non-alcoholic steatohepatitis (NASH), fibrosis and cirrhosis^2^. The pathogenic mechanisms underlying the progression of non-alcoholic fatty liver disease (NAFLD) are not fully understood. Hepatocyte lipoapoptosis (a programmed cell death that is associated with excess lipid accumulation) by free fatty acids (FFAs) is considered to be a key histological feature of NASH and plays a critical role in pathogenesis of NAFLD^3^. The importance of liver apoptosis in NAFLD pathogenesis is strengthened by the evidence that the levels of hepatocyte lipoapoptosis correlates with the disease severity^4^. Proposed mechanisms of lipoapoptosis include induction of ER stress and activation of mitochondrial apoptotic pathway^5^.

Herbal medicine as an alternative approach has long been used to manage various diseases. Herbal medicine is suggested to be a rich source for developing evidence-based chemopreventive or therapeutic agents. Licorice, a popular medicinal plant, has been widely used to treat various diseases including liver disease in China and other Asian countries^6^,^7^. The chemical ingredients of licorice can be divided into four main categories: flavoids, coumarins, triterpenoids and stilbenoids. Glycycoumarin (GCM) is a major coumarin in licorice with favorable pharmacologic property *in vivo*^8^. Previous studies have shown that GCM possesses antiviral^9^,^10^, anti-inflammatory^11^ and anti-spasmodic effect^12^. Our recent study has demonstrated that GCM is able to protect against alcohol-induced hepatotoxicity in both chronic and acute alcoholic liver injury animal models^13^. We hypothesized that GCM could be also effective against non-alcoholic fatty liver disease through suppression of hepatocyte lipoapoptosis. The protective effect of GCM on lipotoxicity has been evaluated using both liver cell culture and methionine/choline-deficient (MCD) diet-induced NASH mouse models. Our results showed that GCM exhibited a strongly inhibiting effect on palmitate-induced lipoapoptosis in the cell culture and a significant reduced hepatotoxicity in the mouse models. Further mechanistic studies revealed that the inhibition of hepatocyte lipoapoptosis by GCM was attributed to its ability to reactivate the impaired autophagy and to suppress ER stress/GSK-3-mediated mitochondrial activation.

## Results

### GCM inhibits palmitate (PA)-induced apoptosis in multiple liver cell lines

**Figure 1:**
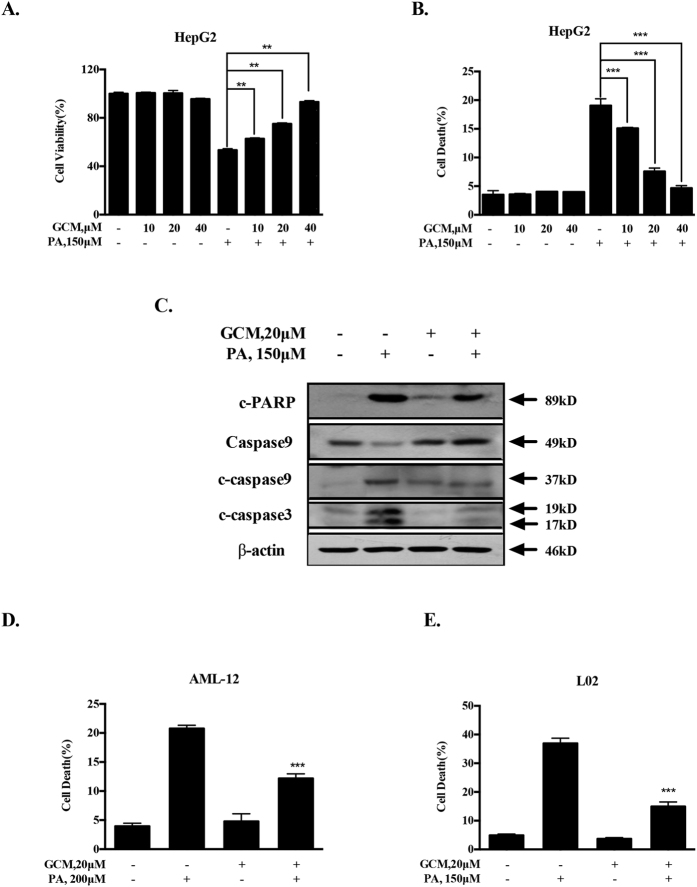
GCM inhibits PA-induced apoptosis in liver cell lines of HepG2, AML-12 and L02. The cells were treated with PA and/or GCM at the indicated concentrations for 24 h and then the cells were harvested for cytotoxicity analysis. (**A)** Preventive effect of GCM on PA-induced reduction of cell viability in HepG2 cells measured by crystal violet staining. (**B**) Inhibitory effect of GCM on PA-mediated apoptosis in HepG2 cells assessed by Annexin V/PI staining. (**C**) Suppression of PA-activated caspases by GCM in HepG2 cells analyzed by western blotting. (**D** and **E**). Protective effect of GCM on PA-mediated apoptosis in AML-12 mouse liver cells (**D**) and in L02 human liver cells evaluated by Annexin V/PI staining (**E**).

To evaluate the protective effect of GCM on PA-induced lipotoxicity, we first measured the changes of cell viability induced by PA in the presence or absence of GCM using crystal violet staining. As shown in Fig. 1A, treatment with GCM alone at concentrations of 10–40 μM did not cause a significant change of cell viability, whereas exposure to 150 μM PA led to a dramatically reduction of HepG2 cell number. In the presence of GCM, the inhibiting effect of PA on cell viability was significantly ameliorated in a concentration-dependent manner. We next employed Annexin v staining to further examine the influence of GCM on PA-induced apoptosis in HepG2 cells. As shown in Fig. 1B, no apoptosis induction was seen in GCM-treated cells, while PA induced a significant increase of apoptosis which was significantly attenuated by co-treating PA with GCM at concentrations of 10–40 μM. The protective effect of GCM on PA-mediated cytotoxicity of HepG2 cells was further validated by the changes of activation of caspases using western blotting. As shown in Fig. 1C, elevated caspase-9/-3 activation and PARP cleavage by PA were reduced by GCM in HepG2 cells. In addition, the protective effect of GCM on lipoapoptosis was also tested in additional liver cell lines. As shown in Fig. 1D and E, similar protective effect of GCM on PA-induced lipotoxicity was also observed in AML-12 mouse liver cells (Fig. 1D) or L02 human liver cells (Fig. 1E).

### GCM protects against hepatocyte lipoapoptosis in MCD diet-induced mouse model of NASH

**Figure 2:**
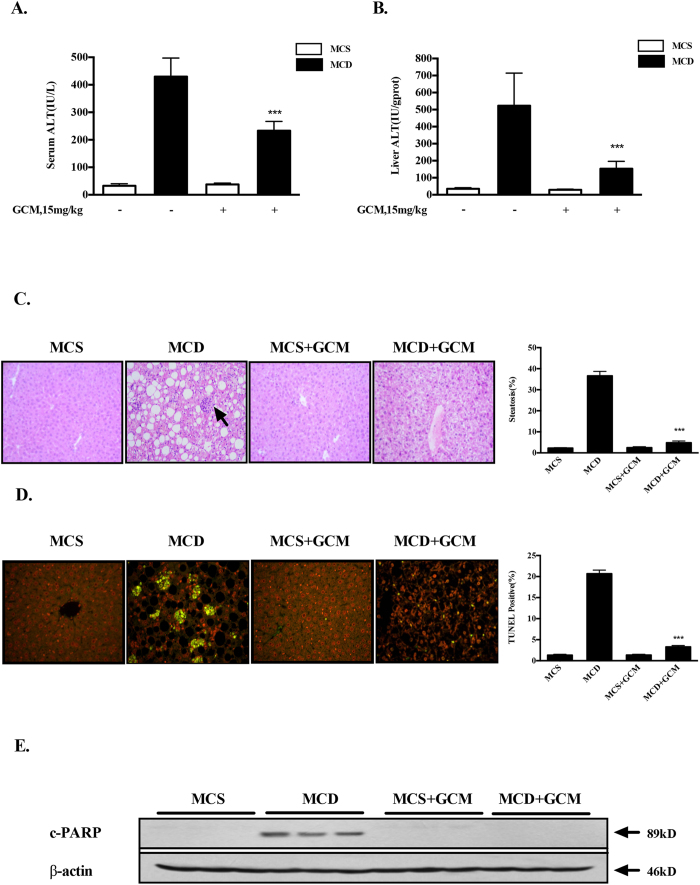
GCM protects against hepatocyte lipoapoptosis in MCD diet-induced mouse model of NASH. Mice were fed MCD or control diet with or without treatment with GCM (15 mg/kg, i.p injection). After 4 weeks, samples were collected for analysis of hepatotoxic markers. (**A** and **B**) Changes of ALT levels in serum and livers measured by a commercial ELISA kit. (**C**) Histological analysis of liver tissues by (**H** and **E**) staining. (**D**) Hepatocyte lipoapoptosis assessed by TUNEL. (**E**) Measurement of PARP cleavage by western blotting.

Having found the inhibiting effect of GCM on PA-induced lipoapoptosis *in vitro*, we asked whether such protective effect can be achieved *in vivo*. To address this issue, C57BL/6 mice were fed with methionine-choline-deficient (MCD) diet for 4 weeks to establish a mouse model of NASH. Biochemical analysis of Alanine aminotransferase (ALT), the key liver injury damage marker, showed that the levels of ALT in both liver and serum were dramatically increased in mice fed with MCD diet compared with that of the control mice. These biochemical changes were significantly reduced in the livers of mice fed with MCD diet plus GCM (Fig. 2A and B). Upon hisological examination, the liver samples of mice fed with MCD diet demonstrated a profound steatosis accompanied by infiltration of inflammatory cells (Fig. 2C). Such pathological features were nearly abolished in the liver samples of mice fed with MCD diet/GCM combination. Consistent with the above biological and pathological results, a significant lipoapoptosis induction was detected in liver samples of mice fed with MCD diet which was almost vanished in the liver samples of mice fed with MCD diet combining with GCM treatment analyzed by TUNEL assay (Fig. 2D). We further validated the inhibiting effect of GCM on hepatocyte lipoapoptosis by western blotting to measure cleavage of PARP, a substrate of caspase. As shown in Fig. 2E, cleavages of PARP were observed in the liver samples of mice fed with MCD diet, but not in the livers of the combination-treated mice. In addition, the body weight reduction in MCD diet-fed mice was partially recovered by the combined treatment (data not shown). Taken together, these results clearly indicate that GCM was able to offer a significant protection against hepatotoxicity in non-alcoholic liver disease animal model via inhibition of hepatocyte lipoapoptosis.

### GCM inhibits activation of mitochondrial pathway

**Figure 3:**
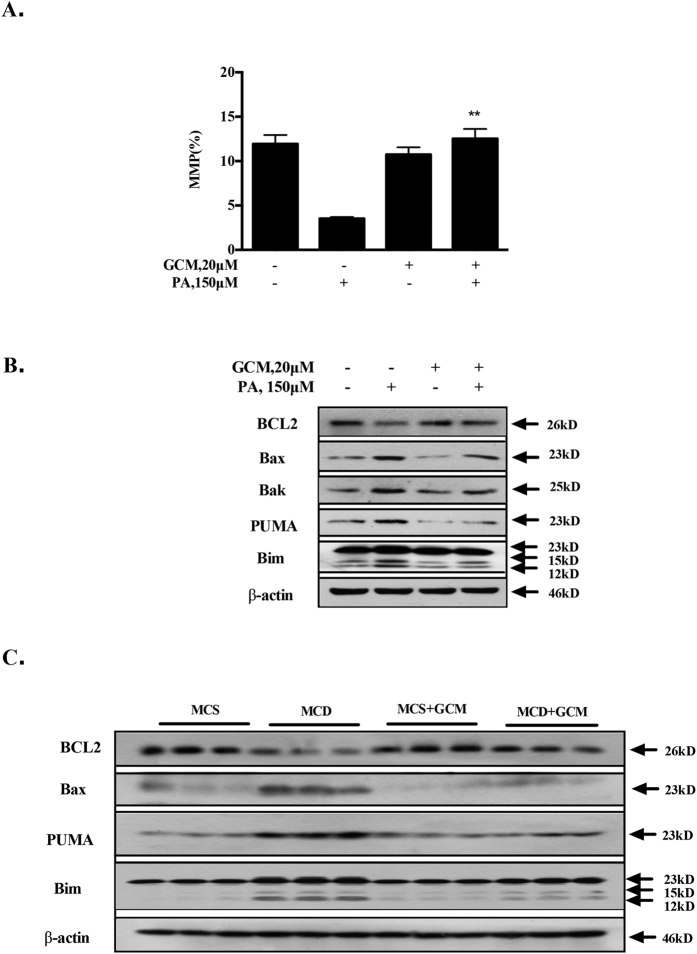
GCM inhibits activation of mitochondrial pathway. The cells were treated with PA and/or GCM at the concentrations indicted for 24 h and then the cell were collected for analysis of mitochondrial pathway. (**A**) Protective effect of GCM on PA-disrupted mitochondrial membrane potential in HepG2 cells determined by JC-1 staining. (**B**) Changes of Bcl-2 family proteins by PA in the presence or absence of GCM analyzed by western blotting in HepG2 cells. (**C**) Influences of GCM on Bcl-2 family protein in liver tissues of mice fed with MCD diet detected by western blotting.

Activation of mitochondrial pathway plays a critical role in PA-mediated lipoapoptosis^14^. Disruption of mitochondrial membrane potential (MMP) is a hallmark of mitochondrial pathway activation. To decipher the mechanisms of the inhibiting effect of GCM on lipoapoptosis, we first investigated influences of GCM on PA-mediated mitochondrial permeability transition. As shown in Fig. 3A, a dramatically decreased MMP was observed in PA-treated cells. In the presence of GCM, this effect was abolished, suggesting that PA-mediated mitochondrial activation was blocked by GCM. Mitochondrial membrane potential is tightly controlled by BCL2 family proteins that have been shown to be involved in PA-mediated hepatocyte lipoapoptosis^14^. We next examined effect of GCM on expressions of BCL2 family proteins. As shown in Fig. 3B, expressions of pro-apoptotic proteins Bax, Bak, PUMA and Bim were increased by PA, whereas expression of anti-apoptotic protein BCL2 was decreased. In the presence of GCM, these changes were significantly attenuated. Furthermore, enhanced expressions of pro-apoptotic BCL2 family proteins and reduction of anti-apoptotic BCL2 family proteins were also found in the livers of mice fed with MCD diet and these pro-apoptotic changes were significantly suppressed by GCM. These results indicated that GCM protected against activation of mitochondrial pathway through regulation of BCL2 family proteins.

### Inhibition of mitochondrial activation by GCM is associated with suppression of JNK and CHOP

**Figure 4:**
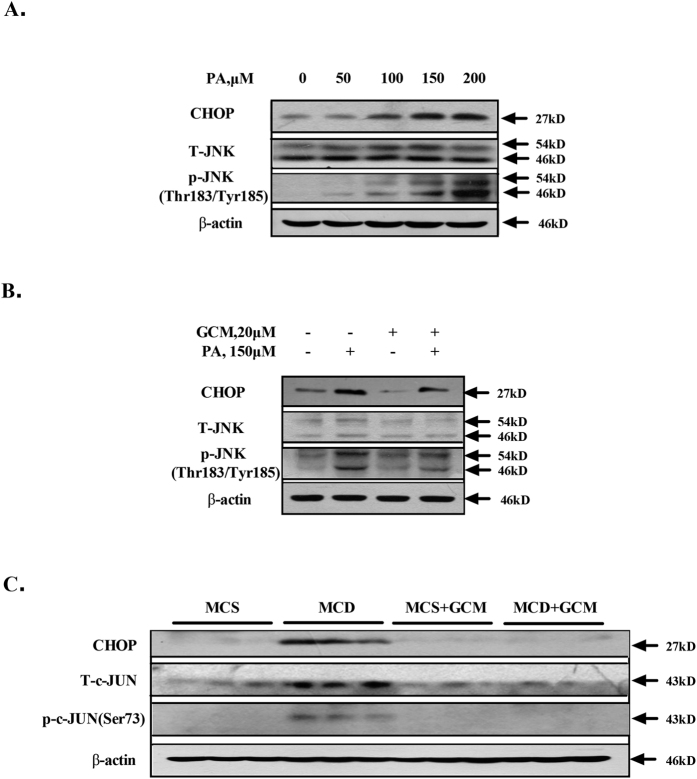
GCM suppresses JNK activation and CHOP induction. (**A**) PA induced a concentration-dependent activation of JNK and CHOP *in vitro*. The cells were treated with the indicted concentrations of PA for 24 h and the phosphorylation of JNK and CHOP expression was examined by western blotting. (**B**) Inhibitory effect of GCM on PA-mediated JNK and CHOP activation *in vitro*. The cells were exposed to PA in the presence or absence of GCM for 24 h and then the changes of JNK phosphorylation and CHOP expression were assessed by western blotting. (**C**) Inactivation of JNK and CHOP by GCM in liver samples analyzed by western blotting.

It has been well documented that JNK and CHOP can trigger mitochondrial-dependent apoptosis through regulation of BCL2 family proteins in certain model systems^15^,^16^. Both JNK and CHOP have been implicated in contributing to PA-induced mitochondrial-mediated lipoapoptosis^17^,^18^. We then asked whether suppression of mitochondrial activation by GCM was attributed to its ability to inactivate JNK and CHOP pathway. We confirmed JNK activation and CHOP induction in PA-treated HepG2 cells (Fig. 4A). In the presence of GCM, PA-mediated activations of JNK and CHOP were significantly reduced (Fig. 4B). Moreover, activations of JNK and CHOP were also found in the livers of mice fed with MCD diet and these activations were clearly ameliorated by GCM (Fig. 4C). These results were well consistent with the changes of their regulated BCL2 family proteins (Fig. 3).

### Suppression of JNK and CHOP by GCM is attributed to the inhibition of ER stress and GSK-3

**Figure 5:**
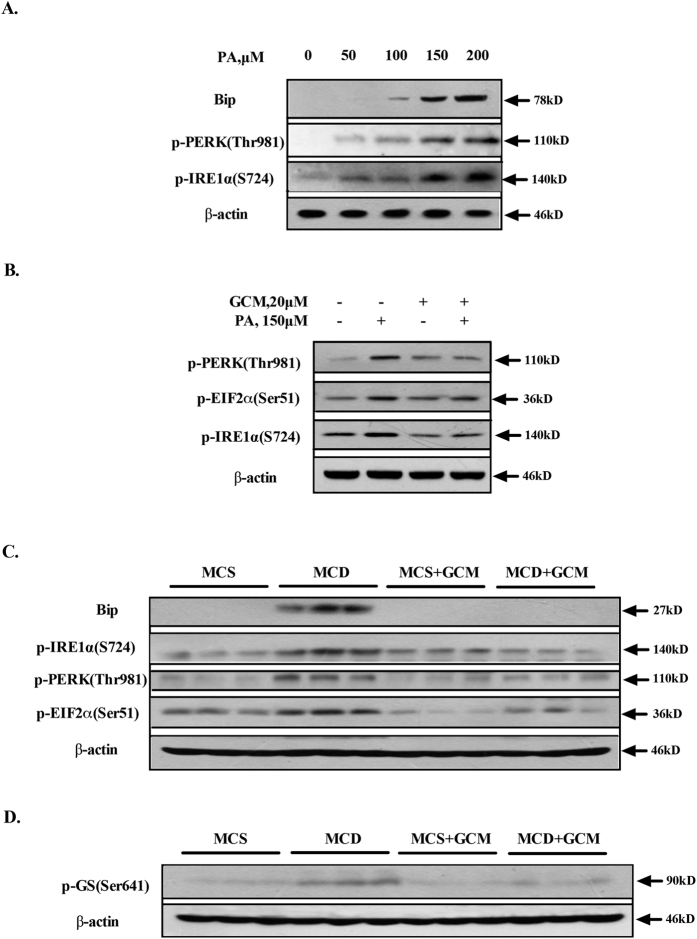
GCM mitigates activation of ER stress and GSK-3. (**A**) PA induced a concentration-dependent activation of ER stress in cell culture model. The cells were treated with the indicted concentrations of PA for 24 h and the key markers of ER stress were examined by western blotting. (**B**) Inhibitory effect of GCM on PA-mediated ER stress in cell culture. The cells were exposed to PA in the presence or absence of GCM for 24 h and then the changes of key ER stress markers were assessed by western blotting. (**C**) Inactivation of ER stress by GCM in liver samples analyzed by western blotting. (**D**) Inhibitory effect of GCM on GSK-3 activation of liver tissues induced by lipid metabolic disorder in MCD diet-mediated NASH model.

Previous studies have demonstrated that both JNK activation and CHOP up-regulation are downstream events of endoplasmic reticulum (ER) stress induced by PA^3^. We therefore explored effect of GCM on PA-activated ER stress. First, PA-induced ER stress was validated in the cell line tested and the results are shown in Fig. 5A. Treatment with PA at concentrations of 50–200 μM caused a concentration-dependent increase of phosphorylation levels of ER resident kinase PERK and IRE1 in HepG2 cells. We next measured the changes of these key ER stress markers in the presence of GCM. As shown in Fig. 5B, the increased phosphorylation levels of PERK and IRE1α were almost abolished by GCM. We further asked whether these *in vitro* findings could be reproducible *in vivo*. As shown in Fig. 5C, a significantly increased protein abundance of ER chaperone Bip as well as the phosphorylation levels of PERK-EIF2α and IRE1α were observed in the livers of mice fed with MCD diet, whereas the induction of these ER stress markers was significantly reduced in the livers of mice fed with MCD diet plus GCM. These results suggested that GCM was able to suppress PA-mediated ER stress both *in vitro* and *in vivo*, which in turn contributing to the inhibition of JNK and CHOP. A recent study by Ibrahim *et al*.^19^ has revealed that Glycogen synthase kinase (GSK)-3 is activated by PA and activation of GSK-3 by PA also contributes to JNK activation. We then investigated effect of GCM on PA-mediated GSK-3 activation. As shown in Fig. 5D, GSK-3 activation (phosphorylated glycogen synthase, p-GS) was indeed enhanced in the liver samples of mice fed with MCD diet, whereas the combination of MCD diet and GCM resulted in a significantly decreased this enhancement action, suggesting that inhibition of GSK-3 maybe also involved in JNK inactivation by GCM.

### Activation of autophagy by GCM contributes to its inhibiting effect on ER stress

**Figure 6:**
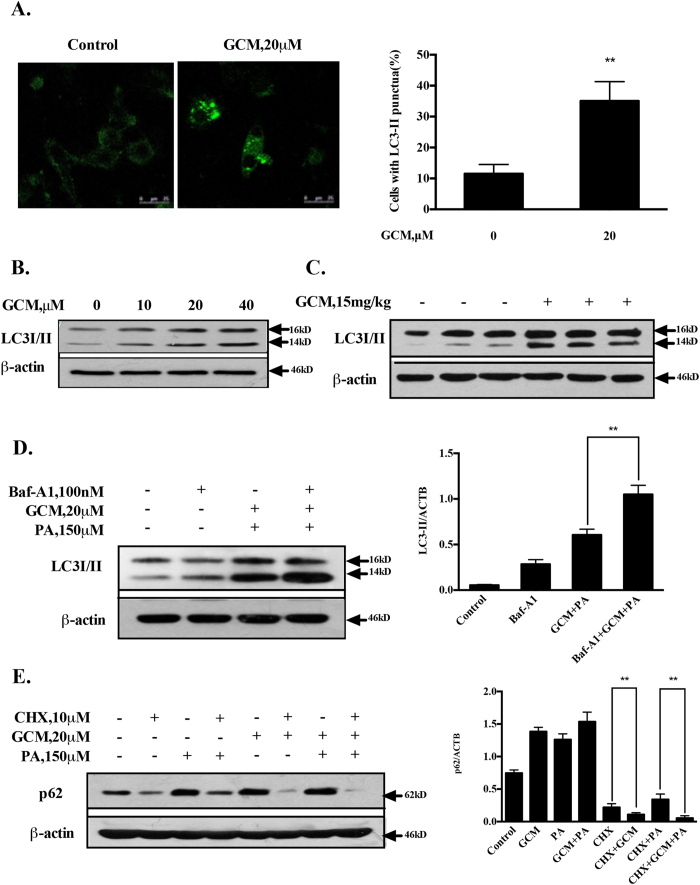
GCM re-activates the impaired autophagy. (**A** and **B**) GCM activated autophagy *in vitro* analyzed by immunofluorescence staining for LC3 distribution or western blotting analysis for conversion of the LC3-I to LC3-II. C. GCM activated autophagy *in vivo* assessed by western blotting. (**D**) GCM increased autophagic flux in the presence of PA. The cells were treated with PA/GCM for 24 h in the presence or absence of 100 nM bafilomycin A1 (added 2 h before cells harvest) and then LC3 was analyzed by western blotting. (**E**). GCM promoted p62 degradation in the presence of PA. The cells were treated with PA and/or GCM in the presence or absence of CHX for 16 h and then p62 was analyzed by western blotting.

**Figure 7:**
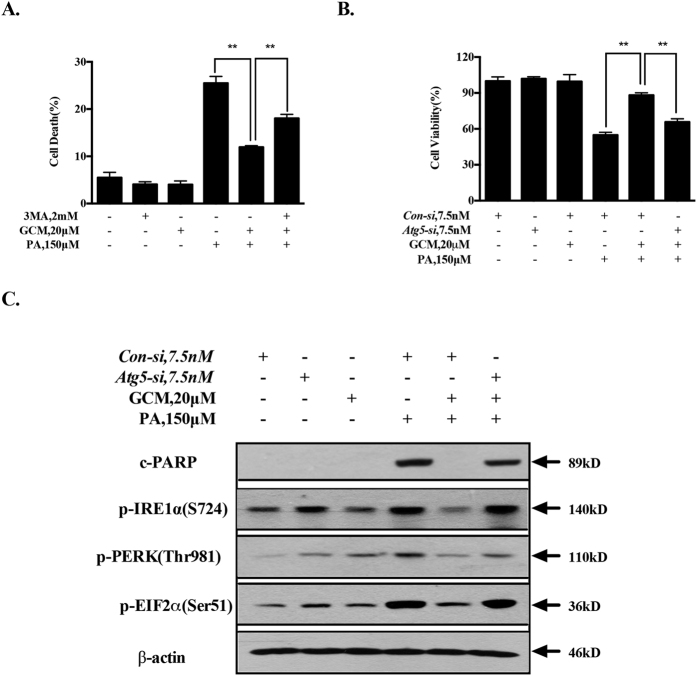
GCM-activated autophagy prevents lipoapoptosis and ER stress in response to PA exposure. (**A** and **B**). Inhibition of autophagy opposed the protective effect of GCM on lipoapoptosis. The cells were treated with PA and/or GCM for 24 h under the condition that autophagy was inhibited by either 3-MA or knockdown of ATG5 and cytotoxicity was evaluated by Annexin V/PI staining (**A**) or crystal violet staining (**B**). (**C**) Inhibition of autopahgy attenuated the suppressing effect of GCM on ER stress. The cells were transfected with ATG5 siRNA or control siRNA for 24 h and then the cells were treated with PA for 24 h and the key ER stress markers were examined by western blotting.

It has been shown that autophagy is impaired in response to PA exposure in cell culture system and in certain animal models of NAFLD^20^. Our recent study has shown that GCM is able to activate autophagy both *in vitro* and *in vivo*^13^. We hypothesized that the impaired autophagy could be restored by GCM, which in turn leading to mitigation of the ER stress. We first validated the activation of autophagy by GCM in the present experimental conditions. As shown in Fig. 6A, a punctated pattern of LC3 fluorescence was detected in GCM-treated cells, which was consistent with the increased conversion of LC3-I to LC3-II (Fig. 6B). Furthermore, this effect was also found in liver tissues of GCM-treated mice (Fig. 6C). We next measured the status of autophagy in response to PA and/or GCM by analyzing autophagic flux. As shown in Fig. 6D, treatment with bafilomycin A1 (BAF), an inhibitor of autophagosome degradation, resulted in increased accumulation of LC3-II owing to autophagy inhibition, whereas LC3-II level was also elevated by PA/GCM combination. Under the condition of autophagy inhibition by BAF, PA/GCM combination caused a further increased LC3-II level, indicating the impaired autophagy was re-activated by GCM. This notion was further verified by measurement of protein levels of p62, a substrate of autophagy that is often used as a marker for autophagy induction^21^. As shown in Fig. 6E, treatment with PA led to an increased p62 owing to autophagy inhibition^20^, whereas p62 was also elevated by GCM. According to our previous study^13^, GCM increases p62 protein level through a mechanism of the transcriptional activation rather than inhibition of its protein degradation. Under the condition that protein translation was blocked by cycloheximide (CHX), a protein synthesis inhibitor, PA still caused an increase of p62, supporting its inhibitory effect on autophagy, but a decreased p62 was observed in GCM-treated cells, suggesting autophagy activation by GCM. Moreover, the reduced p62 level was also found in the combination-treated cells, further suggesting that PA-mediated autophagy inhibition was recovered by GCM. To determine the functional role of autophagy induction in the protective effect of GCM on hepatocyte lipoapoptosis, we assessed influence of autophagy inhibition by a chemical inhibitor or RNAi approach on hepatoprotective effect of GCM. As shown in Fig. 7A and B, when autophagy was inhibited by either 3-MA or siRNA, the protective effect of GCM on lipoapoptosis was significantly compromised. In line with the changes of cytotoxicity, the reduced PARP cleavage and key markers of ER stress by GCM were rebounded (Fig. 7C) under condition of autophagy inhibition by knockdown of ATG5, suggesting involvement of autophagy induction in GCM-mediated suppressive effect on PA-induced ER stress and lipoapoptosis.

## Discussion

Lipoapoptosis is a key event during the progression of NAFLD, targeting lipoapoptosis is therefore a reasonable approach to manage this prevalent liver disease. GCM is a naturally occurring coumarin compound isolated from licorice. In the present study, we have addressed the possibility of the GCM as a chemopreventive agent that protects against hepatocyte lipoapoptosis using *in vitro* and *in vivo* models. We found that hepatocyte lipoapoptosis occurred either in cell culture or animal model was strongly inhibited by GCM. Consistent with lipoapoptosis inhibition by GCM, the other key hepatotoxic markers were also significantly ameliorated. Our findings established the GCM as a novel agent that can prevent NAFLD through suppressing hepatocyte lipoapoptosis.

Induction of ER stress and activation of mitochondrial pathway are considered to be the key mechanisms underlying hepatocyte lipoapoptosis^3^. Activation of JNK and CHOP functions as mediator to convey ER stress signals to mitochondria triggering mitochondrial-dependent apoptosis^22^. In the present study, our results demonstrated that activation of ER stress -JNK/CHOP-mitochondria cascade was inhibited by GCM both *in vitro* and *in vivo*, providing mechanistic support for the protective effect of GCM against hepatocyte lipoapoptosis. In addition to ER stress-mediated JNK activation, activation of GSK-3^19^ and downregulation of Keap1^23^ have been reported to contribute to JNK activation in cell culture system. Our data confirmed the enhanced GSK-3 activation in liver samples of MCD-fed mice and this event was significantly attenuated by GCM, providing additional mechanism of JNK inactivation by GCM. Regarding the role of Keap1, our previous study has shown that Keap1 expression is decreased in response to GCM^13^, suggesting it is unlikely to be involved in GCM-induced JNK inactivation.

Autophagy is a process of the lysosomal degradation of cellular components following multiple forms of cellular stress, such as oxidative stress, ER stress, protein aggregates, damaged organelles and lipogenic challenge^24^,^25^,^26^. The role of ER stress in the regulation of autophagy is well established^27^,^28^. On the other hand, a growing body of evidence suggests that basal autophagy plays a pivotal role in the management of ER stress and maintenance of ER homeostasis^29^,^30^,^31^,^32^. Autophagy deficiency leads to ER stress and loss of ER homeostasis that contributes to the pathogenesis of various diseases^33^. The role of autophagy in the regulation of lipid metabolism remains controversial. A number of studies have shown that autophagy induction can promote degradation of lipid droplets. Activation of autophagy is therefore considered to be an attractive approach to counteract lipid accumulation. For example, a recent study by DeBosch *et al*. demonstrates that trehalose, a naturally occurring disaccharide, induces autophagy and prevents hepatic steatosis^34^. Another study by Narabayashi *et al*. also shows that autophagy activation by indomethacin offers a cytoprotective effect that may be attributed to autophagy-mediated degradation of lipid droplets (lipophagy)^35^. In contrast, a study by Kim *et al*. shows that autophagy deficiency in skeletal muscle leads to a decreased fat mass and a low hepatic lipid content^36^. The mechanisms behind these conflicting effects of autophagy on lipid metabolism remain unclear. Autophagy inhibition has been reported in PA-treated liver cells and HFD or MCD-induced animal model of NASH^20^. More importantly, impaired autophagic flux was also observed in NASH patients^20^,^23^. Inhibition of basal autophagy is suggested to play critical role in activation of ER stress and induction of lipoapoptosis. Recovery of autophagic flux by rapamycin, a well-known autophagy inducer, indeed prevents ER stress and lipoapoptosis in response to palmitate exposure^20^, suggesting activation of autophagy is a practical approach to inhibit hepatocyte lipoapoptosis. Our present data revealed that inhibited autophagy by PA was re-activated by GCM evidenced by decreased p62 level and increased autophagic flux. Functional analysis of autophagy activation revealed that autophagy played a role in GCM-mediated inactivation of ER stress, inhibition of lipoapoptosis and prevention of steatosis in MCD mouse model, suggesting a beneficial role of autophagy induction by GCM in the regulation of hepatic lipid metabolism and in the suppression of ER-stress-mediated lipoapoptosis. Further study is clearly needed to investigate the detailed mechanisms underlying GCM-induced autophagy-mediated lipid metabolism. Our findings provided a new support for autophagy as a target to fight against NAFLD.

Lipoapoptosis and lipid accumulation are the key features of MCD-induced liver injury. Proposed mechanisms of this model include autophagy defect, ER stress^20^, increased fatty acid uptake and decreased VLDL secretion^37^. In the present study, we mainly addressed the role of autophagy activation in the inhibition of lipoapoptosis by GCM and additional mechanisms contributed to suppression of steatosis by GCM are being investigated. In addition, even through MCD mouse model is a commonly used NASH animal model, it has certain disadvantages. For instance, MCD-fed mice display loss of bodyweight and decreased serum levels of insulin and leptin^38^. Validation of the protective effect of GCM on lipoapoptosis in additional animal models is needed for future study.

**Figure 8:**
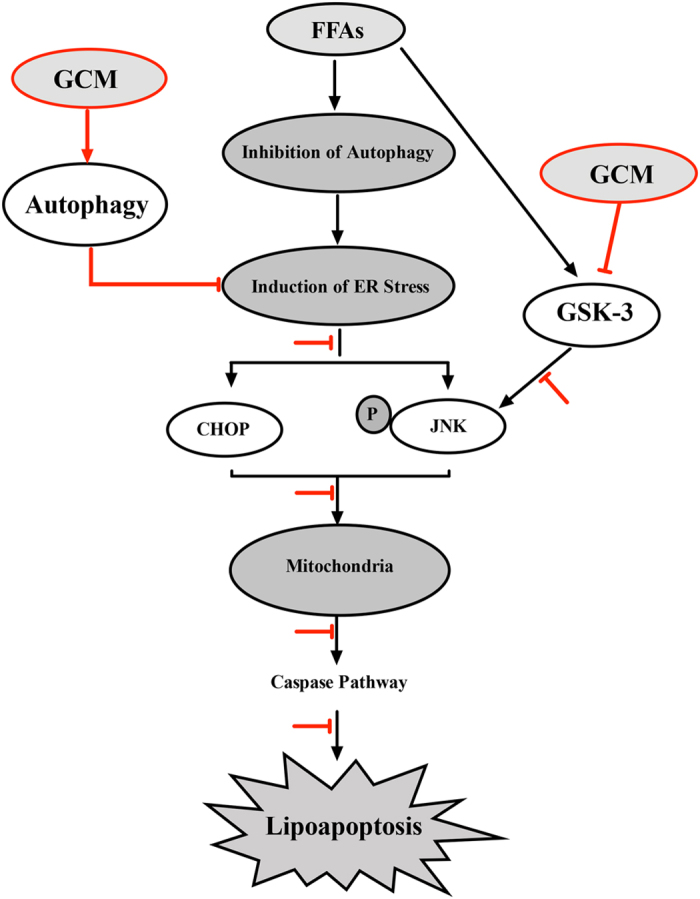
Potential signaling pathways underlying the inhibitory effect of GCM on hepatocyte lipoapoptosis. Lipid metabolic disorder impairs the basal autophagy, leading to activation of ER stress-JNK-mitochondrial pathway. In the presence of GCM, the impaired autophagy is reactivated and subsequently the activated ER stress and its downstream events are inhibited. In addition, GSK-3 is activated by lipid metabolic disorder, which also contributes to activation of JNK-mitochondrial pathway. These events are suppressed by GCM. Together, GCM protects against hepatocyte lipoapoptosis via activation of autophagy and inhibition of ER stress/GSK-3-JNK-mitochondial cascade.

In summary, our present study has identified GCM as a novel ingredient that contributes to hepatoprotective effect of licorice against NAFLD. GCM is able to offer protection against hepatocyte lipoapoptosis through activation of autophagy and mitigation of ER stress/GSK-3 –JNK/CHOP-mitochondria cascade activation (Fig. 8).

## Materials and Methods

### Chemicals and reagents

Glycycoumarin (GCM purity >99%) was purchased from BioBioPha. Sodium palmitate (P9767), cycloheximide (CHX) (C1988) and 3-MA (M9281) were purchased from Sigma-Aldrich. Primary-antibodies specific for Bip (3183), phospho-EIF2α (3597), phospho-PERK (3192), CHOP (2895), Bax (2772), Bak (12105), BCL2 (3869), PUMA (14570), Bim (2933), c-PARP (9548), caspase 9 (9502), c-caspase 3 (9929), c-caspase 9 (9929), phospho-GS (3891) and p-JNK (4668) were purchased from Cell Signaling Technology. Phospho-IRE1α (ab48187) was purchased from Abcam. Primary-antibody specific for β-actin (AT0001) was purchased from Action Biotech. Primary-antibodies specific for p62 (PM045), LC3 (PM036) and horseradish peroxidase-conjugated secondary antibodies were purchased from MBL International Corporation. Mitochondrial membrane potential assay kit with JC-1 (C2006) was purchased from Beyotime.

### Cell culture and treatments

HepG2 cells were grown in Dulbecco’s Modification of Eagle’s Medium (DMEM) (Thermo, SH30022.01B) supplemented with 10% fetal bovine serum without antibiotics. AML-12 cells were grown in Dulbecco’s Modification of Eagle’s Medium: F12(DMEM:F12 1:1;Thermo,SH30023.01) supplemented with 10% fetal bovine serum without antibiotics. L02 cells were grown in RPMI-1640 Medium (Thermo, SH30809.01) supplemented with 10% fetal bovine serum without antibiotics. At 12–24 h after plating when cells were 50–60% confluence, the medium was changed before starting the treatment with GCM and/or other agents.

### Crystal violet staining

For the assessment of cell viability, after treatment, the culture medium was removed and the cells were fixed in 1% glutaral-dehyde solution in PBS for 15 min. The fixed cells were stained with 0.02% aqueous solution of crystal violet for 30 min. After washing with PBS, the stained cells were solubilized with 70% ethanol. The absorbance at 570 nm with the reference filter 405 nm was evaluated using a microplate reader (Thermo).

### Apoptosis evaluation

Apoptosis was determined by flow cytometry following Annexin V/PI double staining of externalized phosphatidyl-serine (PS) and DNA fragmentation in apoptotic cells using Annexin V/PI staining kit from MBL International Corporation, or by western blot analysis cleavage of PARP1, a substrate of caspase.

### Measurement of mitochondrial membrane potential

Mitochondrial membrane potential was detected by flow cytometry following JC-1 staining.

### Western blotting

The cells were lysed with ice-cold RIPA (radio-immuno-precipitation assay) buffer. Equal amount of proteins of the samples were loaded onto the gel. After electrophoretic separation, the proteins were transferred to a nitrocellulose membrane. The membrane was subsequently probed with primary antibodies followed by incubation with corresponsive secondary antibody. The immune-reactive blots were visualized using enhanced chemi-luminescence (Fisher/Pierce, Rockford, IL, USA) and recorded on X-ray film (Eastman Kodak Company, Rochester, NY, USA).

### RNA interference

ATG5 siRNA(AM16708) and negative control siRNA(AM4641) were purchased from Ambion. The cells were transfected with 7.5 nM of specific or negative control siRNA using INTERFER in siRNA transfection reagent according to the manufacturer’s instructions (Polyplus-Transfection, Inc. 409–10). 24 h post-transfection, the cells were used for subsequent experiments.

### Autophagy detection

Autophagy induction was determined by two methods. The first method was western blotting analysis for conversion of the LC3-I to LC3-II or changes of autophagy substrate p62. The second one was immunofluorescence staining for LC3 distribution.

### Animal and diets

Animal Care and experimental protocols were approved by the Institutional Animal Care and Use Committee (China Agricultural University). The experiments were carried out in accordance with the approved guidelines. Male 8-week-old C57BL/6 mice (Charles River, Beijing) were divided into four groups (n = 8) and fed with a methionine/choline-sufficient (MCS) diet, a methionine/choline-deficient (MCD) diet, a MCS diet plus GCM (15 mg/kg) and a MCD diet plus GCM (15 mg/kg). GCM was dissolved with 5% tween 80 and injected intraperitoneally daily. After 4 weeks, animals were sacrificed for the collection of plasma and liver tissue samples. Plasma was stored at −80 °C and portions of liver tissue were fixed in neutral buffered formalin, whereas others were frozen immediately in liquid nitrogen and stored at −80 °C for further analysis.

### Biochemical assay

ALT was measured using a kit from Nanjing Jiancheng Bioengineering Institute (Nanjing, China).

### Histochemical and immunehistochemical staining

Liver tissue was stained with hematoxylin and eosin (H&E). Terminal deoxynucleotidyl transferase-mediated dUTP nick end labeling assay (TUNEL, Termo G3250) was employed to examine the lipoapoptosis in mouse liver tissues, according to the manufacturer’s instructions.

### Statistical analysis

Data were presented as mean ± SD. These data were analyzed by the ANOVA followed with Bonferroni-Dunn post hoc test. p < 0.05(*) was considered statistically significant.

## Additional Information

**How to cite this article**: Zhang, E. *et al*. Glycycoumarin inhibits hepatocyte lipoapoptosis through activation of autophagy and inhibition of ER stress/GSK-3-mediated mitochondrial pathway. *Sci. Rep.* **6**, 38138; doi: 10.1038/srep38138 (2016).

**Publisher's note:** Springer Nature remains neutral with regard to jurisdictional claims in published maps and institutional affiliations.
